# Betulin inhibits inflammatory factors synthesis in osteoarthritis synovial fibroblasts by suppressing the PI3K, Akt, and mTOR pathways and activating miR-5006-5p

**DOI:** 10.7150/ijms.130493

**Published:** 2026-03-17

**Authors:** Kun-Tsan Lee, Chun-Hao Tsai, Yu-Han Wang, Xiu-Yuan He, Yi-Chin Fong, Chih-Yuan Ko, Chih-Hsin Tang

**Affiliations:** 1 Department of Post-Baccalaureate Medicine, National Chung-Hsing University, Taichung, Taiwan.; 2 Department of Orthopedics Surgery, Taichung Veterans General Hospital, Taichung, Taiwan.; 3 Department of Orthopedic Surgery, China Medical University Hospital, Taichung, Taiwan.; 4 Department of Sports Medicine, College of Health Care, China Medical University, Taichung, Taiwan.; 5 Department of Pharmacology, School of Medicine, China Medical University, Taichung, Taiwan.; 6 Department of Orthopedic Surgery, China Medical University Beigang Hospital, Yunlin, Taiwan.; 7 Department of Medical Laboratory Science and Biotechnology, College of Medical and Health Science, Asia University, Taichung, Taiwan.; 8 Chinese Medicine Research Center, China Medical University, Taichung, Taiwan.

**Keywords:** Betulin, Osteoarthritis, CHI3L1, ICAM-1

## Abstract

Osteoarthritis (OA) is a widespread joint condition often linked to aging and obesity, resulting in pain, joint dysfunction, and disability. Betulin, a lupane-type pentacyclic triterpene alcohol extracted from birch trees, exhibits anti-inflammatory properties; however, its anti-inflammatory effects in OA remain largely unknown. Our high-throughput cytokine array data exhibit that betulin inhibits two key inflammatory factors, CHI3L1 and ICAM-1, in OA synovial fibroblasts (OASFs). Results from the GEO database and our clinical tissues confirm that CHI3L1 and ICAM-1 levels are markedly higher in OA patients compared to healthy individuals. Furthermore, anterior cruciate ligament transection (ACLT)-induced OA rats exhibited upregulated CHI3L1 and ICAM-1 expression. Mechanistically, we demonstrated that betulin inhibits CHI3L1 and ICAM-1 synthesis in OASFs by inhibiting the PI3K, Akt, and mTOR pathways and activating miR-5006-5p. Importantly, molecular docking analysis predicted an interaction between betulin with CHI3L1 and ICAM-1, suggesting its direct effects. Our investigation suggests that betulin is a leading candidate for OA management.

## Introduction

As a result of medical progress and rising life expectancy, degenerative illnesses have emerged as some of the majority frequent health concerns, with osteoarthritis (OA) being one of the most widespread 1. The Global Burden of Disease report indicated that around 528 million people globally had OA, with its prevalence rising by 114.5% from 1990 to 2019 2. Disease-specific features of OA comprise subchondral bone breakdown, cartilage erosion, and inflammation of the synovial tissue 3. These features are mostly irreversible by the time of diagnosis and lead to stiffness and joint pain 4. At present, OA cannot be cured, and the available treatment options are restricted to slowing down the progression of the disease or easing pain 5.

Joint pain, structural damage, and the release of synovial fluid are closely linked to chronic inflammation of the synovial tissues in OA, where the latter plays a crucial role in driving tissue breakdown and inflammation 6. Chitinase 3-like 1 (CHI3L1) is a chitinase-like protein without enzymatic activity that is presented extensively in prokaryotic and eukaryotic cells 7. It has been identified in a range of cell types, such as fibroblast, epithelial, cartilage and bone 8. Accumulating studies show that CHI3L1 remains present under chronic inflammatory conditions and affects cell differentiation, proliferation and apoptosis when stimulated by inflammatory cytokines during arthritis 9, 10. Alongside inflammation, as OA progresses, intercellular adhesion molecule-1 (ICAM-1) is one of numerous adhesion molecules that regulate the attachment of monocytes to the synovium 1. It has been demonstrated that synovial tissue from OA patients produces a higher quantity of ICAM-1 than that from healthy individuals 11. As a result, scientists have recognized the suppression of ICAM-1 production in the synovium as a potential approach to alleviate arthritis-associated inflammation 12.

Synthetic molecules and natural compounds based on natural prototypes have received growing attention owing to their low toxicity and biological functions 13-15. Betulin, a lupane-type pentacyclic triterpene derived from birch tree bark, has attracted attention for its pharmacological properties, which include a diverse array of actions for instance anti-inflammatory, antibacterial, and antioxidant effects 16-18. Studies have shown that it could possess therapeutic qualities for addressing various ailments, including diabetes, cancer, and inflammatory diseases 19, 20. Betulin has been associated with anti-inflammatory properties due to its ability to control immune activations and block the synthesis of pro-inflammatory cytokines 21. In arthritis, betulin has been documented to block inflammatory cytokines IL-1β and TNF-α generation 20. However, the functions of betulin in OA treatment remain poorly understood. Our high-throughput cytokine array data exhibit that betulin inhibits both CHI3L1 and ICAM-1 production in OA synovial fibroblasts (OASFs). Inhibition of the PI3K, Akt, and mTOR pathways, along with activation of miR-5006-5p, contributes to betulin-inhibited effects. Interestingly, computational modeling predicts that betulin likely interacts with CHI3L1 and ICAM-1. Thus, betulin represents a promising novel therapeutic agent for OA.

## Material and Methods

### Materials

The antibodies against mTOR (SC-136369), p-p85 (SC-12929), p85 (SC-1637), p-Akt (SC-16646-R), Akt (SC-5298) and the p85 activator (SC-3036) were purchased from Santa Cruz Biotechnology (Santa Cruz, CA). The antibodies against ICAM-1 (4915S) and p-mTOR (5536S) were acquired from Cell Signaling (Danvers, MA, USA). CHI3L1 antibody (GTX639462) was acquired from GeneTex (Hsinchu, Taiwan). The mTOR activator (MHY1485) was purchased from MedChemExpress (NJ, USA). Betulin (B9757) and the Akt activator (SML079) were obtained from Sigma-Aldrich (St. Louis, MO).

### Cell culture

OASFs were obtained from the synovial tissues with OA through collagenase treatment, following previously described methods 22. OA specimens were digested using 3% collagenase (C6885, Sigma-Aldrich) in serum-free DMEM (with antibiotics) for 4-6 hrs. The suspension was subsequently centrifuged at 1000 rpm for 10 mins. After removed supernatant, the medium was replaced with DMEM (with 10% FBS and antibiotics). This procedure was repeated every 24 hours until no further cell detachment was observed. All cells were kept in DMEM (with 10% FBS and antibiotics) within a 5% CO_2_ incubator at 37°C.

### Human clinical samples

Patients undergoing knee arthroplasty at China Medical University Hospital provided the human synovial samples that were collected. This research included tissues collected from ten patients with end-stage osteoarthritis and six patients who had arthroscopic examinations due to trauma-related conditions. The Institutional Review Board of China Medical University Hospital approved the research protocol. All patients provided written informed consent prior to their inclusion in the study 23.

### Bioinformatic analysis

CHI3L1 and ICAM-1 levels were analysed using a dataset GSE89408 that was taken from the Gene Expression Omnibus (GEO) database 24. For ''core analysis'', we selected differentially expressed genes from dataset GSE55457 that had logFC values of ≥1 and ≤-1 to input into the QIAGEN Ingenuity Pathway Analysis (IPA) system. After the expression analysis was selected, genes from the Ingenuity Knowledge Base were utilized to enhance the canonical signaling pathways.

### RT-qPCR

RNA was isolated using TRIzol reagent (MDBio Inc., Taipei, Taiwan), and 1 μg of RNA was converted to cDNA with the M-MLV reverse transcription kit (Thermo Fisher Scientific, Waltham, MA). Quantitative PCR amplification was conducted with the StepOnePlus™ Real-Time PCR System and gene-specific primers (MDBio Inc.). The level of fold changes in target genes was compared to the internal control gene GAPDH and calculated using the 2⁻ΔΔCt formula 14, 25.

### Western blot analysis

Cells were lysed in 100 μL RIPA buffer supplemented with protease inhibitors (Roche, Indianapolis, IN, USA). Equal protein amounts (30 μg/lane) were separated on 10% SDS-PAGE gels and transferred onto PVDF membranes. The membranes were blocked with 5% fat-free milk prepared in TBS for 1 hour under room temperature conditions to reduce the binding of antibodies to unspecific protein binding sites. Following blocking, membranes were left to react with primary antibodies overnight at 4 °C. After washes, treatment with HRP-conjugated secondary antibodies occurred. Protein bands were visualized with the use of ECL reagents and imaged with the iBright 1500 Imaging System (Thermo Fisher Scientific, Waltham, MA). Densitometry was assayed with ImageJ software (NIH, Bethesda, MD, USA) 26, 27.

### Protein array analysis

Total protein was assayed using Human XL Cytokine Array Kit (ary022b, Thermo Fisher Scientific, Waltham, MA). Membranes were initially blocked with blocking buffer in a 4-well multi-dish for 1 hour. For sample incubation, 100 µL of cell lysis buffer was diluted to a total volume of 1.5 mL and incubated with the membranes overnight at 2-8°C. Following the primary incubation and subsequent washes, the membranes were treated with an antibody cocktail for 1 hour under continuous agitation. After further washing steps, the membranes were incubated with 1X streptavidin-HRP for 30 minutes. Finally, the membranes were washed again and evenly covered with a chemiluminescent reagent for signal detection. Protein spots were visualized with the use of ECL reagents.

### Luciferase reporter assay

Following the transfection of the cells with either the wild-type (WT) or mutant (MT) CHI3L1 and ICAM-1-3'-UTR luciferase plasmid employing Lipofectamine 2000. After 24 hours of transfection, the cells were lysed and luciferase activity was immediately assessed with the Dual-Luciferase® Reporter Assay System (Promega Corporation). To account for transfection efficiency, Firefly luciferase signal was normalized against Renilla luciferase signal.

### Anterior cruciate ligament transection (ACLT)-induced OA model

Six male Sprague-Dawley rats, weighing 300-350 g, were purchased from BioLASCO Taiwan Co., Ltd. (Taipei, Taiwan). Rats were anesthetized by intraperitoneal injection of Zoletil® 50 (20-40 mg/kg) combined with xylazine (5-10 mg/kg), and ACLT surgery was performed 25, 28. Prior to the surgical procedure, each rat was administered a preoperative intraperitoneal injection of cefazolin (20 mg/kg), with the same dosage repeated every two days during the postoperative period. Following standard shaving and disinfection, the right knee joint was accessed via a medial parapatellar approach. With the knee positioned in full flexion, the patella was dislocated laterally to facilitate the transection of the anterior cruciate ligament using sterile scissors. Immediately after surgery, rats received a subcutaneous injection of the analgesic lidocaine (7 mg/kg). The surgical site was disinfected with povidone-iodine. Ten weeks after surgery, rats were euthanized in a CO₂ chamber with a gradual fill rate of 30-70% chamber volume per minute for 4 minutes. All animal experiments were performed in strict accordance with the 3R principles and were approved by the Institutional Animal Care and Use Committee of China Medical University.

### Staining with immunohistochemistry (IHC)

Anti-CHI3L1 and ICAM-1 antibodies were utilized to stain slices of synovial tissue. Utilizing techniques outlined in our earlier work 29, staining results were measured. In short, the tissues were applied with primary antibodies. Binding of the secondary antibody and DAB staining were carried out with a Leica Novolink Polymer Detection system. The final staining scores were calculated by summing the intensity and percentage scores 14, 23.

### Statistical analysis

The mean ± standard deviation (SD) is used to display the data. The two-tailed Student's t-test was used to assess statistical significance between experiment groups. One-way ANOVA was used for comparisons involving more than two groups, and Tukey's post hoc test was used to assess the significance of group differences at a significance level of 0.05.

## Results

### Betulin inhibits CHI3L1 and ICAM-1 production in OASFs

**Figure 1:**
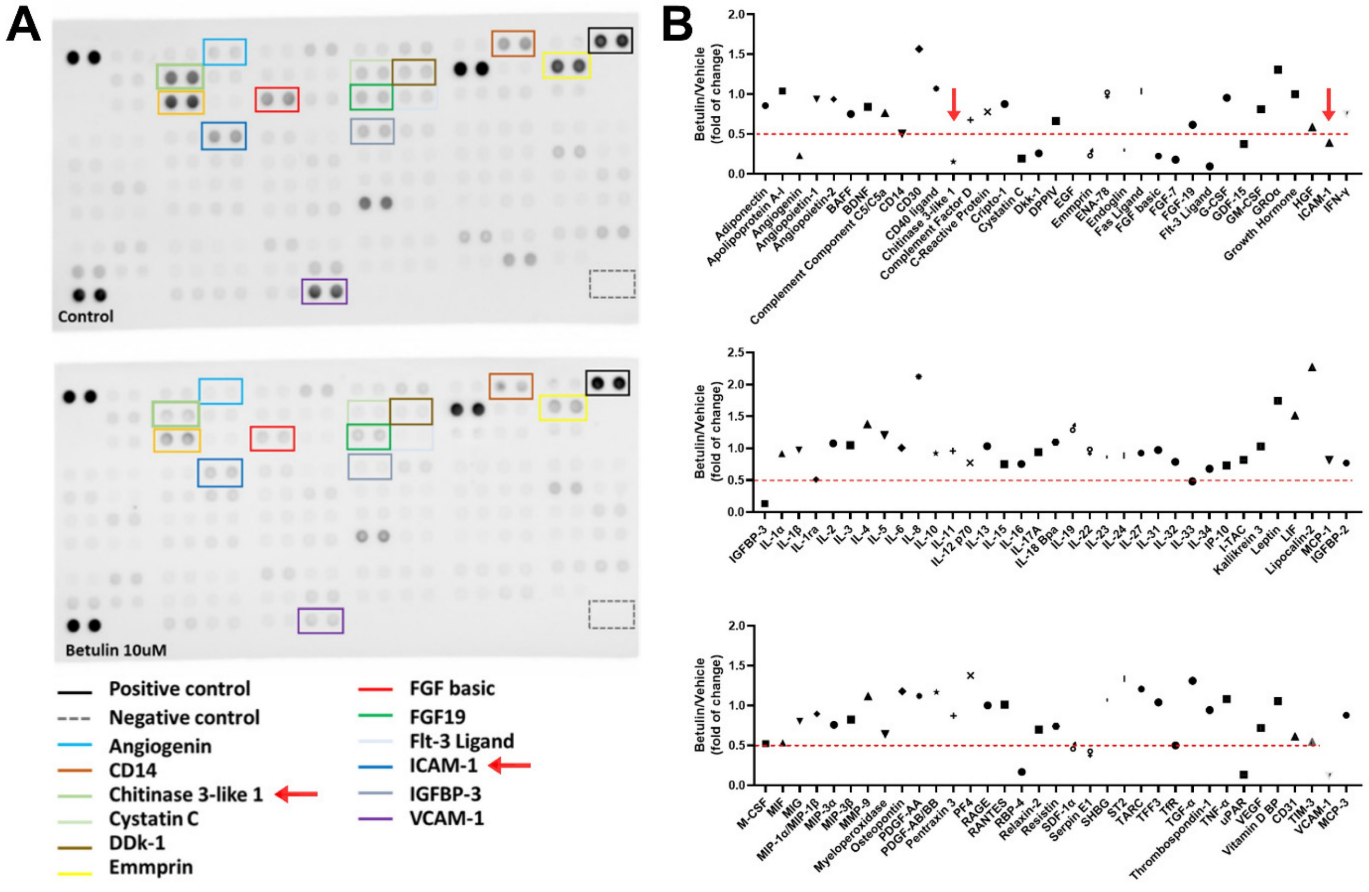
** Betulin inhibits inflammatory factors production in OASFs.** (A&B) Results of cytokine array displaying expression of inflammatory factors by OASFs applied with betulin for 24 h.

**Figure 2:**
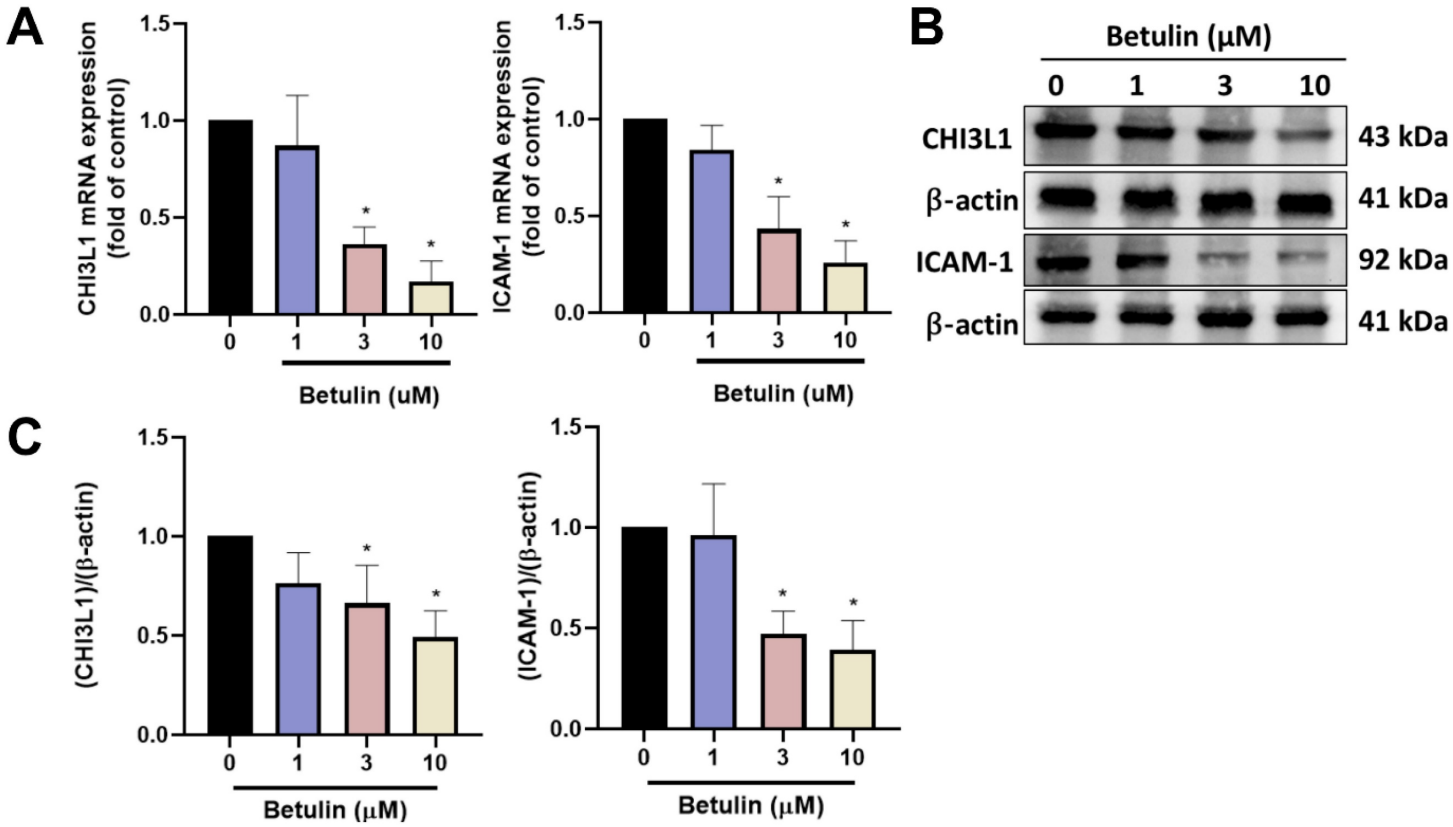
** Betulin inhibits CHI3L1 and ICAM-1 expression in OASFs.** (A) OASFs were applied with betulin for 24 h, the indicated mRNA expression was examined by qPCR. (B&C) OASFs were applied with betulin for 24 h, the CHI3L1 and ICAM-1 expression was examined by Western blotting. *p < 0.05 compared with the control group. ^#^*p* < 0.05 compared with the betulin-treated group.

**Figure 3:**
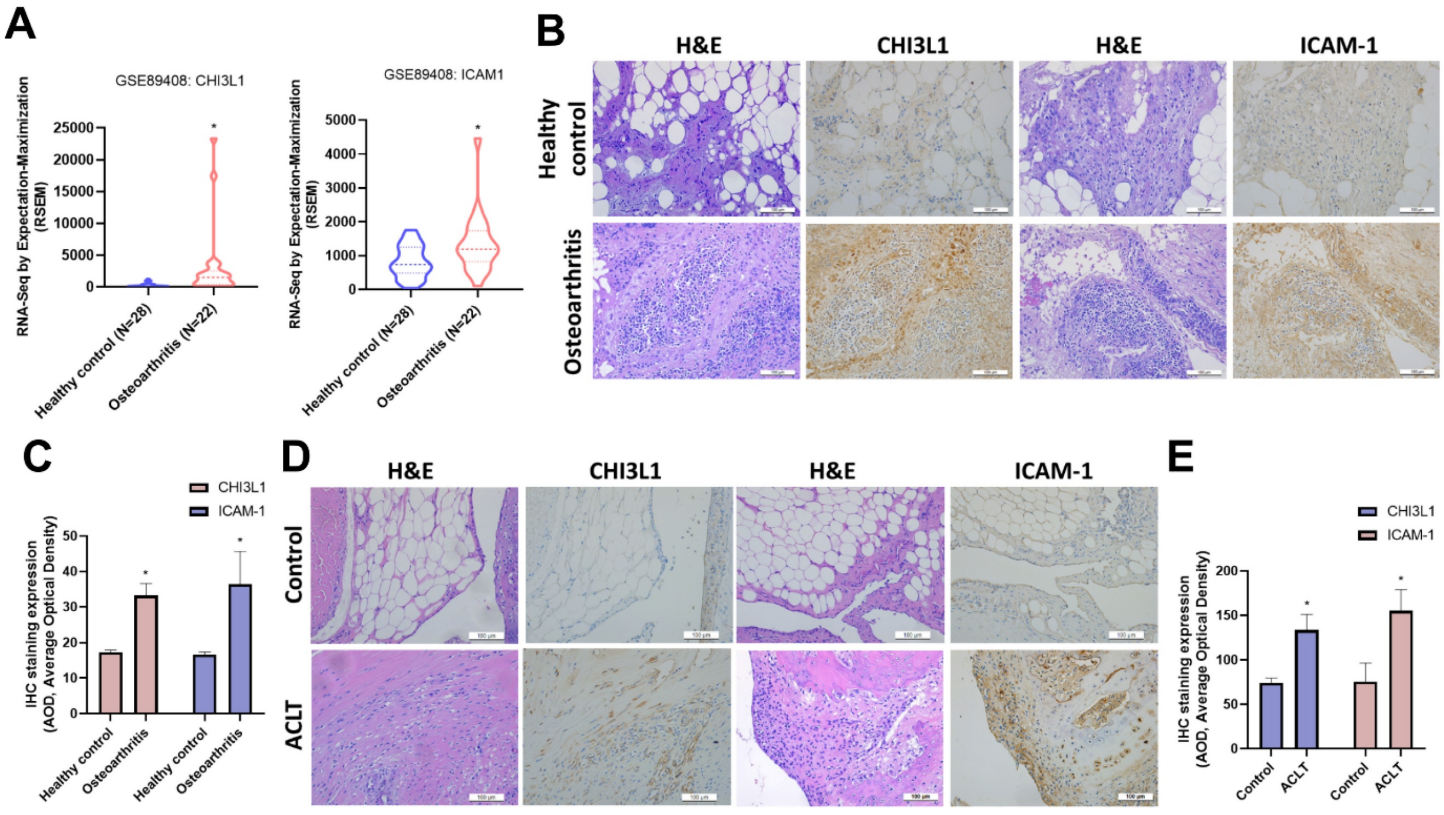
** CHI3L1 and ICAM-1 expression levels are higher in patients with OA.** (A) The levels of CHI3L1 and ICAM-1 determined using the GSE89408 dataset. (B&C) H&E staining and IHC staining of CHI3L1 and ICAM-1 in synovial tissues from normal controls and OA patients. (D&E) IHC staining of CHI3L1 and ICAM-1 in synovial tissues from control and ACLT rats. *p < 0.05 compared with the control group.

Betulin exhibits several biological functions, including anti-inflammatory properties 20. We therefore examined the functions of betulin in cytokine production in OASFs. High-throughput cytokine array data exhibited that betulin strongly downregulated CHI3L1 and ICAM-1 expression (Fig. 1A&B). Furthermore, OASFs treated with betulin markedly diminished CHI3L1 and ICAM-1 mRNA synthesis in a concentration-dependent manner (Fig. 2A). Additionally, betulin reduced CHI3L1 and ICAM-1 protein production in OASFs (Fig. 2B&C). Data from the GEO database also confirm that CHI3L1 and ICAM-1 are upregulated in OA patients compared to healthy controls (Fig. 3A). Our clinical sample data also show similar results, with upregulated CHI3L1 and ICAM-1 in OA patients (Fig. 3B&C). The ACLT model is a well-established animal model for inducing OA 30. Notably, ACLT-induced OA rats exhibited upregulated CHI3L1 and ICAM-1 expression (Fig. 3D&E). Thus, betulin inhibits CHI3L1 and ICAM-1 synthesis in OASFs, which in turn reduces OA progression.

### Betulin suppresses CHI3L1 and ICAM-1 expression through the PI3K, Akt and mTOR pathways

**Figure 4:**
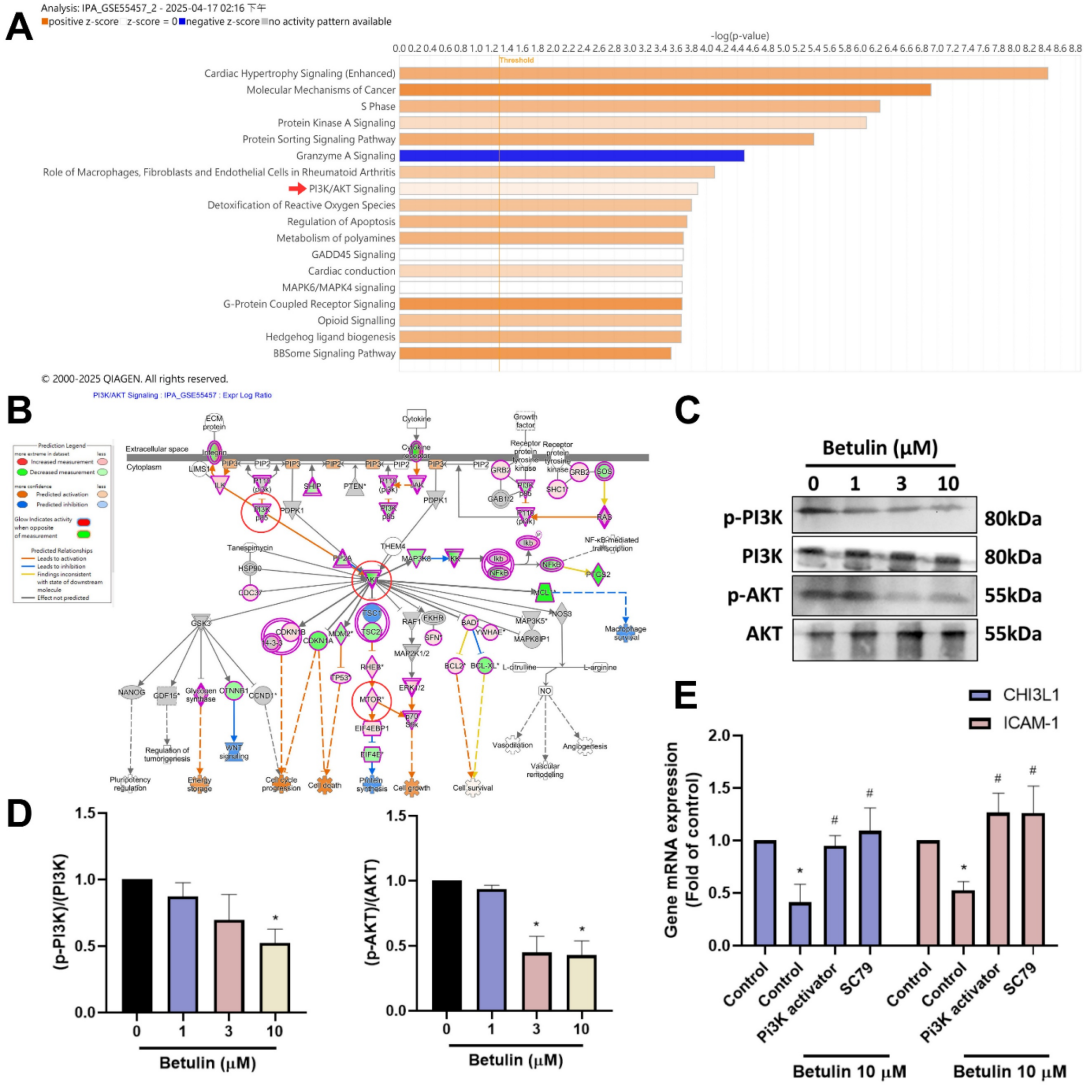
** The PI3K and Akt pathways are involved in the inhibitory effects of betulin.** (A&B) The GSE55457 dataset's altered pathways are displayed in the IPA pathway enrichment figure. (C&D) OASFs were applied with betulin, the PI3K and Akt phosphorylation was examined by Western blotting. (E) OASFs were applied with PI3K and Akt activators and then treated with betulin for 24 h, the indicated mRNA expression was examined by qPCR. *p < 0.05 compared with the control group. ^#^*p* < 0.05 compared with the betulin-treated group.

**Figure 5:**
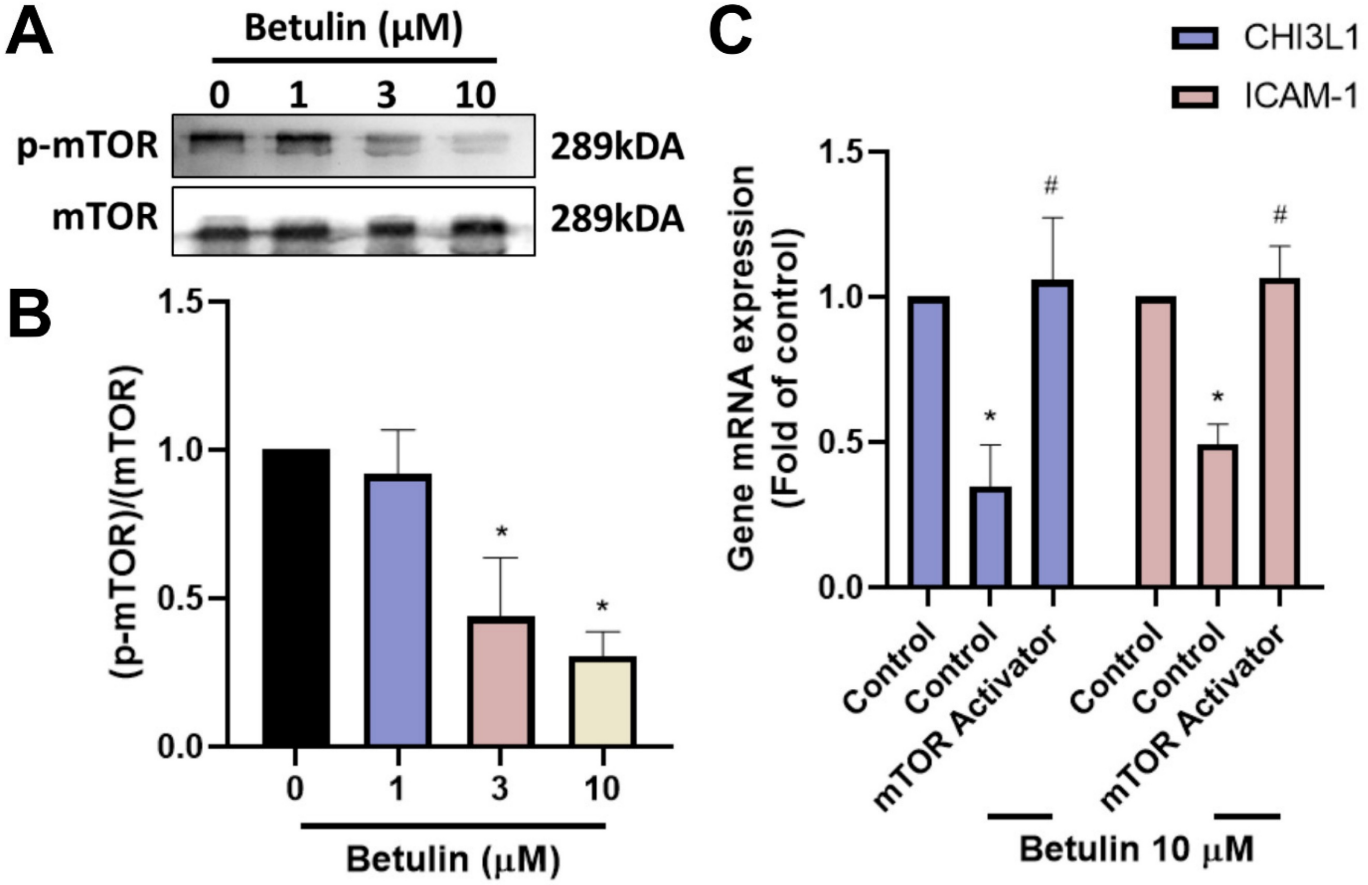
** The mTOR pathway is involved in the inhibitory effects of betulin.** (A&B) OASFs were applied with betulin, the mTOR phosphorylation was examined by Western blotting. (C) OASFs were applied with mTOR activator and then treated with betulin for 24 h, the indicated mRNA expression was examined by qPCR. *p < 0.05 compared with the control group. ^#^*p* < 0.05 compared with the betulin-treated group.

To discovery the signaling pathways controlled in the regulation of OA progression, we investigated IPA analysis on the GSE55457 dataset. The analysis found that the PI3K, Akt and mTOR signaling mechanisms were linked to the top PI3K and Akt signaling in OA progression (Fig. 4A&B). We next performed Western blotting to investigate the function of PI3K, Akt and mTOR signaling pathways following betulin application. The results showed that betulin inhibits phosphorylation of PI3K and Akt (Fig. 4C&D). To confirm that PI3K and Akt mediate betulin's inhibitory effects, pharmacological activators were used. Treatment with PI3K and Akt (SC79) activators blocked the betulin-mediated inhibition of CHI3L1 and ICAM-1 expression (Fig. 4E). Similarly, stimulation of OASFs with betulin diminished mTOR phosphorylation (Fig. 5A&B). The mTOR activator also antagonized betulin-reduced CHI3L1 and ICAM-1 expression (Fig. 5C). These findings indicate that betulin reduces CHI3L1 and ICAM-1 synthesis through the PI3K, Akt and mTOR signaling pathways.

### miR-5006-5p regulates the expression of CHI3L1 and ICAM-1 inhibited by betulin

**Figure 6:**
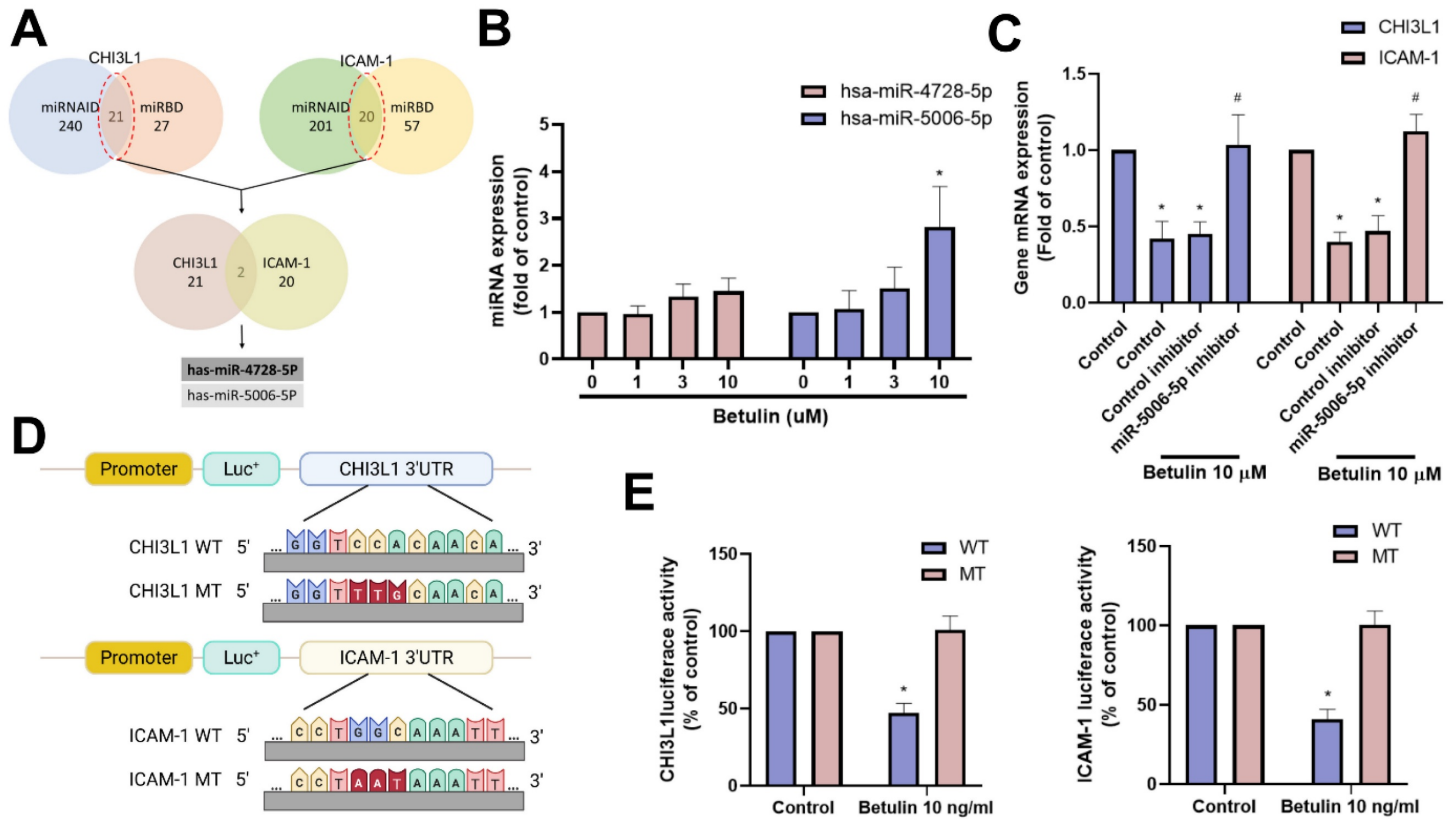
** miR-5006-5p controls betulin-inhibited CHI3L1 and ICAM-1 expression.** (A) The diagrams illustrate the selection of miRNA candidates aimed at CHI3L1 and ICAM-1. (B) OASFs were treated with betulin for 24 h, the indicated miRNA expression was examined by qPCR. (C) OASFs were transfected with treated with miR-5006-5p inhibitor then applied with betulin for 24 h, the miR-5006-5p expression was examined by qPCR. (D&E) OASFs were transfected with indicated luciferase plasmids then with betulin, the 3'UTR activity was examined by luciferase activity. *p < 0.05 compared with the control group. ^#^*p* < 0.05 compared with the betulin-treated group.

miRNAs are small non-coding RNAs that regulate several pathological functions in arthritis 31, 32. Employing two bioinformatics tools (miRNAID and miRBD), we uncovered two miRNAs that directly interact with the 3' UTRs of both CHI3L1 and ICAM-1 (Fig. 6A). Betulin treatment primarily promoted miR-5006-5p but not miR-4728-5p expression (Fig. 6B). Further experiments were carried out to ascertain if betulin inhibits CHI3L1 and ICAM-1 by regulating miR-5006-5p. These involved transfecting OASFs with a miR-5006-5p inhibitor, which blocked the roles of betulin on CHI3L1 and ICAM-1 production (Fig. 6C). To assess the effect of miR-5006-5p on CHI3L1 and ICAM-1 gene transcription (Fig. 6D), luciferase reporter plasmids were utilized that included the WT-CHI3L1 and ICAM-1 3'-UTRs, as well as regions with MT predicted for miR-5006-5p. In WT-CHI3L1 and ICAM-1 3'-UTR plasmids, betulin triggered luciferase activity, whereas it did not do so in MT-CHI3L1 and ICAM-1 3'-UTR plasmids (Fig. 6E). This indicates that betulin diminishes CHI3L1 and ICAM-1 by promoting miR-5006-5p synthesis.

### Forecasted docking conformations suggest that betulin binds to CHI3L1 and ICAM-1

**Figure 7:**
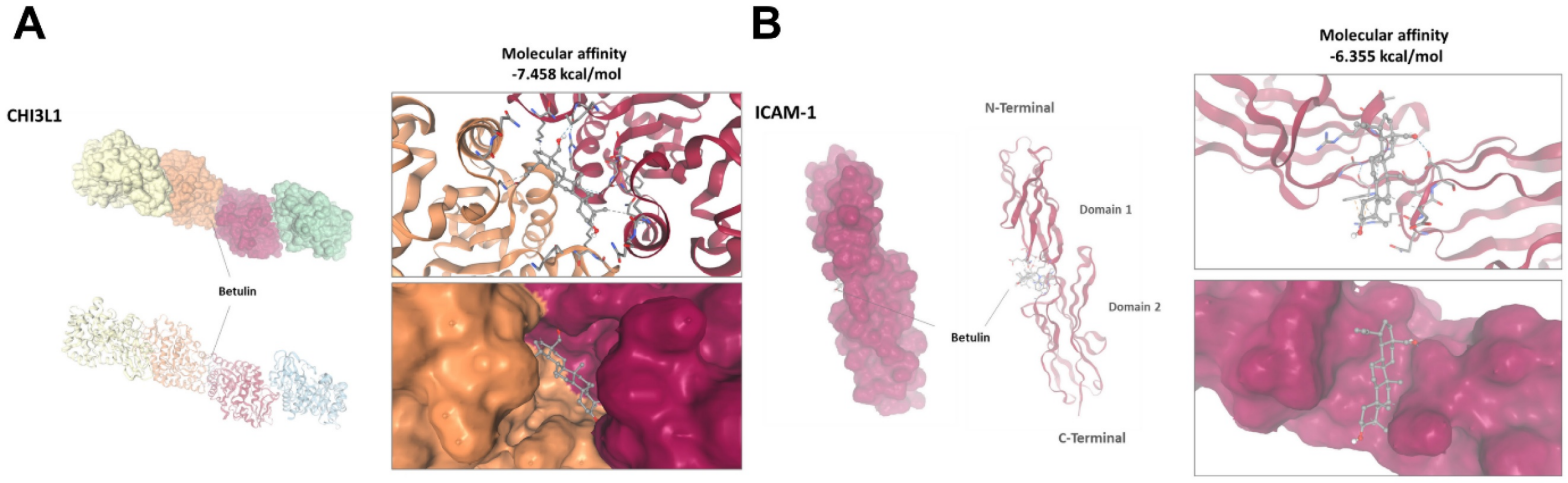
** Predicted molecular interaction between betulin with CHI3L1 and ICAM-1.** (A&B) Molecular docking analysis showing the predicted binding of betulin with CHI3L1 and ICAM-1.

We next investigated if betulin can access intracellular targets by traversing cell membranes. In order to examine this possible interaction, we anticipated the binding model of betulin with CHI3L1 and ICAM-1. The anticipated docking conformations of betulin with the CHI3L1 and ICAM-1 proteins are depicted in Figure 7. Betulin demonstrated a higher binding affinity, with predicted docking energies of -7.458 kcal/mol for CHI3L1 and -6.355 kcal/mol for ICAM-1 (Fig. 7A&B).

## Discussion

The choric inflammatory nature of OA causes irreversible damage to the afflicted joints, and the exact etiology of inflammatory system dysfunction is still unknown 33. Common characteristics include immune cell infiltration and synovial inflammation. Activated synovial fibroblasts facilitate cartilage deterioration and joint inflammation by generating proinflammatory mediators. This adds to the OA synovial microenvironment's "vicious cycle" of disease progression 34. As an experimental cell model, we employed OASFs. A high-throughput cytokine array exhibited CHI3L1 and ICAM-1 as the most potent cytokine inhibited by betulin stimulation in OASFs. We also demonstrated that betulin reduces CHI3L1 and ICAM-1 production in OASFs by inhibiting the PI3K, Akt, and mTOR pathways and activating miR-5006-5p. Importantly, molecular docking studies indicate that betulin directly binds to CHI3L1 and ICAM-1. The betulin may serve as a potential therapeutic agent for OA remedy.

The PI3K and Akt signaling mechanisms are crucial for a wide range of biological process, such as differentiation, motility and apoptosis 35, 36. Our IPA analysis of the GSE55457 dataset revealed that the PI3K, Akt and mTOR signaling mechanisms were linked to the top PI3K and Akt signaling in OA progression. We demonstrate that PI3K and Akt activators blocked betulin-induced inhibition of CHI3L1 and ICAM-1 expression. Additionally, betulin administration diminished the phosphorylation of PI3K and Akt. The PI3K and Akt cascades, which activate mTOR, are closely linked to cellular functions, establishing them as essential components of the cell signal transduction network 36, 37. Our results using a pharmacological activator targeting mTOR demonstrate that mTOR regulates betulin-mediated reduction of CHI3L1 and ICAM-1 production. Betulin application suppresses mTOR phosphorylation, indicating that the PI3K, Akt, and mTOR pathways are controlled in betulin-promoted inhibition of CHI3L1 and ICAM-1 synthesis in OASFs.

Through fully complementary or imperfect base-pairing, miRNAs bind to the 3'-UTR of their corresponding mRNAs, resulting to a repression of translation or a reduction in the stability of the bound mRNAs 38, 39. The examination of miRNA database software conducted in this investigation found that miR-5006-5p and miR-4728-5p interrupts CHI3L1 and ICAM-1 transcription. Later investigations demonstrated that the administration of betulin resulted in the promotion of miR-5006-5p and miR-4728-5p expression in OASFs. Furthermore, introducing an inhibitor of miR-5006-5p into OASFs counteracted the CHI3L1 and ICAM-1 production inhibited by betulin. The 3'-UTR luciferase reporter assay demonstrated that miR-5006-5p directly binds to the 3'-UTR of CHI3L1 and ICAM-1, regulating their gene transcription. This indicates that betulin modulates CHI3L1 and ICAM-1 production in OASFs by increasing miR-5006-5p levels. However, we did not examine whether miR-5006-5p also interacts with the PI3K, Akt, and mTOR pathways. Further investigation should examine in detail the interaction between miR-5006-5p and the PI3K, Akt, and mTOR pathways.

Many studies have recorded heightened CHI3L1 synthesis in the synovial fluid and cartilage of those suffering from OA, indicating a strong link between CHI3L1 and arthritis 10. Prior studies concentrated on CHI3L1 as an arthritis biomarker, aiming to utilize CHI3L1 as a diagnostic marker for OA 40, 41. On the other hand, the migration of monocytes from blood vessels to sites of inflammation heavily depends on the extracellular domain of ICAM-1 34. In the synovium of OA individuals, ICAM-1 up-regulation has been demonstrated, suggesting it may play a significant role in the recruitment of monocytes to the synovial tissue 12. Moreover, it is proposed that lowering the levels of ICAM-1 in synovial fluid would be an effective means of curbing the inflammatory activity and alleviating symptoms of physiological distress in OA 42, 43. In the current study, we used a computational model to predict whether betulin has a direct role in interacting with CHI3L1 and ICAM-1. Our molecular docking analysis predicted an interaction between betulin and CHI3L1 and ICAM-1, with docking energies of -7.458 kcal/mol for CHI3L1 and -6.355 kcal/mol for ICAM-1. These results indicate that betulin regulates CHI3L1 and ICAM-1 production not only through cellular mechanisms but also by directly binding to CHI3L1 and ICAM-1. This limitation should be acknowledged. In the present study, we did not provide direct experimental evidence of these interactions and relied exclusively on predicted binding models. Future studies should investigate whether betulin directly binds to CHI3L1 and ICAM-1.

**Figure 8:**
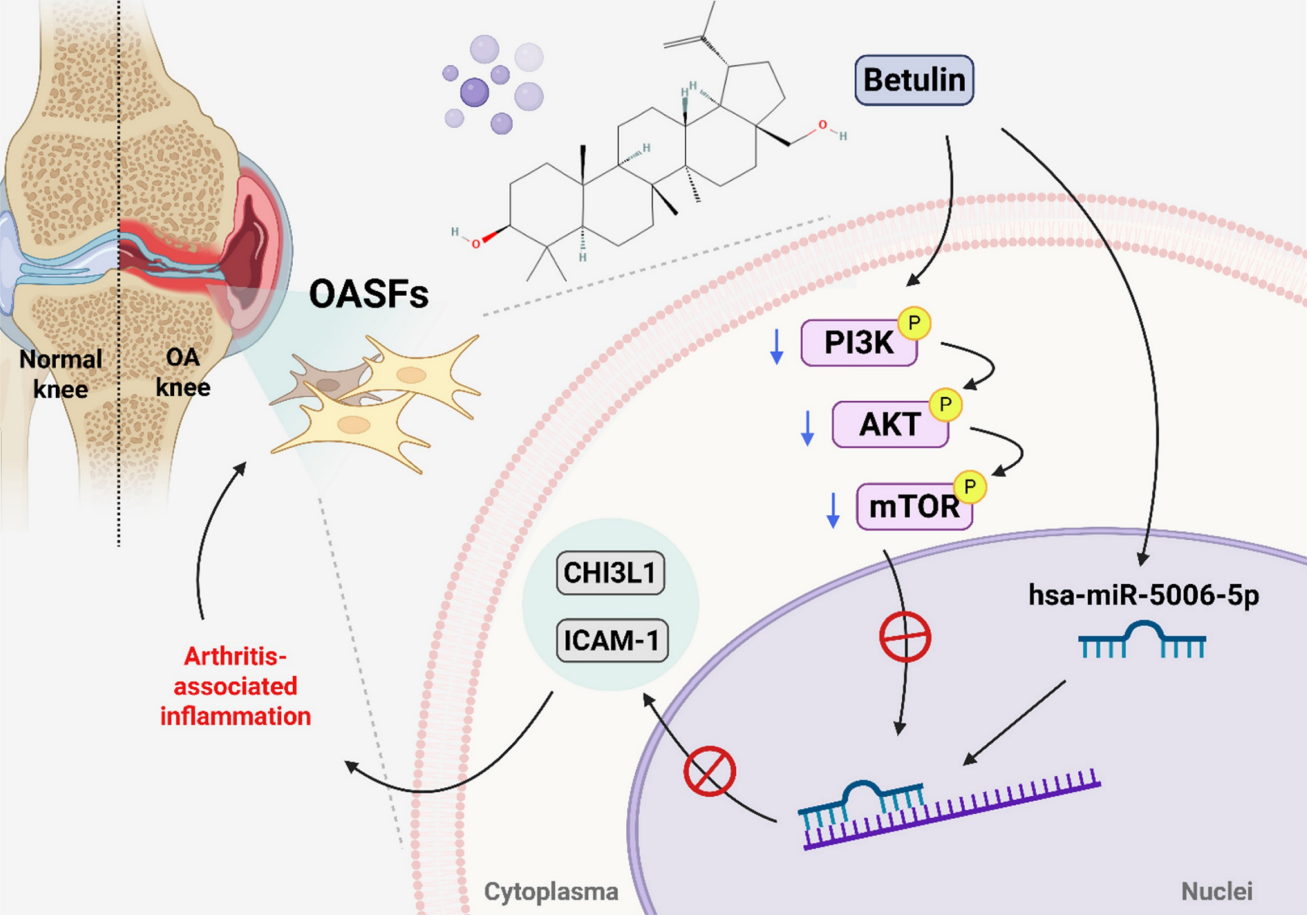
** Schematic diagram illustrating the mechanism underlying the CHI3L1 and ICAM-1 production of betulin in OA progression.** Betulin inhibits CHI3L1 and ICAM-1 synthesis in OASFs by inhibiting the PI3K, Akt, and mTOR pathways and activating miR-5006-5p, which inhibits OA progression.

In conclusion, we demonstrate that betulin inhibits CHI3L1 and ICAM-1 synthesis in OASFs by inhibiting the PI3K, Akt, and mTOR pathways and activating miR-5006-5p (Fig. 8). Molecular docking results indicate an interaction between betulin with CHI3L1 and ICAM-1. Betulin may serve as a potential therapeutic agent for OA treatment.
